# Measurement of Temperature and Relative Humidity with Polymer Optical Fiber Sensors Based on the Induced Stress-Optic Effect

**DOI:** 10.3390/s18030916

**Published:** 2018-03-20

**Authors:** Arnaldo Leal-Junior, Anselmo Frizera-Neto, Carlos Marques, Maria José Pontes

**Affiliations:** 1 Graduate Program of Electrical Engineering of Federal University of Espirito Santo, 29075-910 Vitória, Brazil; frizera@ieee.org (A.F.-N.); mjpontes@ele.ufes.br (M.J.P.); 2 Instituto de Telecomunicações, Campos Universitário de Santiago, 3810-193 Aveiro, Portugal; carlos.marques@ua.pt

**Keywords:** polymer optical fiber, temperature sensor, relative humidity sensor, stress-optic effect

## Abstract

This paper presents a system capable of measuring temperature and relative humidity with polymer optical fiber (POF) sensors. The sensors are based on variations of the Young’s and shear moduli of the POF with variations in temperature and relative humidity. The system comprises two POFs, each with a predefined torsion stress that resulted in a variation in the fiber refractive index due to the stress-optic effect. Because there is a correlation between stress and material properties, the variation in temperature and humidity causes a variation in the fiber’s stress, which leads to variations in the fiber refractive index. Only two photodiodes comprise the sensor interrogation, resulting in a simple and low-cost system capable of measuring humidity in the range of 5–97% and temperature in the range of 21–46 °C. The root mean squared errors (RMSEs) between the proposed sensors and the reference were 1.12 °C and 1.36% for the measurements of temperature and relative humidity, respectively. In addition, fiber etching resulted in a sensor with a 2 s response time for a relative humidity variation of 10%, which is one of the lowest recorded response times for intrinsic POF humidity sensors.

## 1. Introduction

Humidity and temperature monitoring are important in applications such as structural health monitoring (SHM) and pharmaceutical, medical, and food processing and storage [1,2]. For example, in wearable robotics, the measurement of temperature and humidity is applied to monitor the microclimate conditions between human skin and the robotic device. The microclimate assessment is very important to prevent injuries due to skin maceration [3]. Furthermore, adverse microclimate conditions cause discomfort and may cause the user to abandon such technology. Although the human body can tolerate a wide range of temperatures and humidity, there are three general regions of human comfort that have been defined [3]. These regions are as follows: (i) comfort in temperatures in the range of 29–34 °C with relative humidity (*RH*) below 70%; (ii) neutral comfort in relative humidity below 80% and temperatures between 27 and 36 °C; and (iii) discomfort in temperatures lower than 27 °C or higher than 36 °C or in relative humidity higher than 80% [3]. Therefore, temperature and humidity sensors suitable for wearable robotics should be able to operate within these defined regions of comfort.

Conventional technologies for humidity measurement include the use of materials that contract or expand with variations in humidity. However, the materials’ variations are slow and nonlinear [2]. Wet and dry bulb psychrometers consist of two thermometers measuring the dry and wet bulb temperatures, from which the *RH* is estimated. Although this method provides a reliable measurement with a low-cost system, it cannot be applied in small or enclosed areas [2], such as in the case of microclimate sensing. Electronic sensors with capacitive and resistive transducers are widely used; however, they may present a response time of longer than 30 s and can suffer from electromagnetic interference [3], which inhibits their application to wearable robots. This electromagnetic interference is also a significant limitation for conventional technologies for temperature measurement such as thermocouples, thermistors, and resistance-based temperature detectors [4].

Optical fibers have well-known advantages such as compactness, lightness of weight, multiplexing capabilities, and electromagnetic immunity [5] that enable them to be used as sensors for different parameters. One of these parameters is *RH*, and several techniques have been proposed throughout the years. Such techniques include resonant frequency-based sensors [6], Mach–Zehnder interferometers [7], etched fiber-based sensors [8], and fiber Bragg gratings (FBGs) [9], among others. Some of these approaches are used for temperature measurement as well [10,11,12]. However, these methods generally need complex signal processing. In addition, the implementation and expense of the interrogation equipment can make these technologies unsuitable for low-cost applications [13]. 

For humidity sensing, silica optical fiber-based sensors need to be in contact with a sensing material, which can be gelatin films, modified claddings, polymeric coatings, or different dopants [2]. This, however, can reduce sensor reproducibility, because the process of applying the sensing material may vary, which leads to variations in sensor behavior and increases the difficulty of sensor manufacturing. Furthermore, some of these sensors work on a limited range of *RH* and can also present a longer response time in some cases [2]. For these reasons, the discussion in this paper is limited to intrinsic humidity sensors.

Additional advantages of polymer optical fiber (POF) include higher flexibility, higher fracture toughness, and better biocompatibility [14]. There are different materials that may comprise POFs, which include TOPAS [15], Zeonex [16], polycarbonate [17], CYTOP [18], and poly (methyl methacrylate) (PMMA) [19]. PMMA fibers present humidity sensitivity due to their water absorption [14], which enables these types of POFs to act as intrinsic humidity sensors. In addition, PMMA POFs with a larger diameter (about 1 mm) are widely available commercially, and they may be employed with low-precision plastic connectors, which generally result in a lower-cost system [20]. Moreover, due to their higher numerical aperture, low-cost lasers or light emitting diodes (LEDs) [21] may also be used. Woyessa et al. [22] used a polymer optical fiber Bragg grating (POFBG) to measure humidity, which operates stably in a humidity range of 10–90% and in temperatures between 25 and 75 °C. POFBGs for moisture and humidity measurements have also been presented in [23,24]. However, these sensors need high-cost interrogation systems. Rajan et al. [25] presented a POFBG on PMMA POF to measure humidity, and in order to enhance the sensor’s response time, an etching was made on the fiber, which resulted in one of the lowest response times for intrinsic humidity sensors based on POFs. Regarding low-cost options for humidity sensing, Muto et al. [26] presented a POF humidity sensor based on power attenuation, which is a low-cost interrogation method that provides a fast response. However, this sensor requires the use of a sensitive clad, which increases the sensor’s fabrication difficulty and affects its reproducibility.

In order to overcome some of the limitations of humidity sensors and to achieve a system capable of measuring climate parameters, this paper presents the development of a low-cost system for measuring temperature and humidity with POF sensors. The sensor is based on variations in the fiber’s mechanical properties due to temperature and humidity variations. If the fiber is submitted to predefined torsional stress, the variation in its mechanical properties leads to a variation in the fiber’s output power due to the stress-optic effect. Such effect is the variation in the fiber’s refractive index when it is submitted to stress [27]. Furthermore, as presented in [24], stress applied on the fiber can reduce the response time of the sensor. Moreover, fiber etching further reduces the sensor’s response time.

This paper is organized as follows. Section 2 describes the operation principle of the proposed sensors. Section 3 presents the experimental setup employed for the sensor characterization. Section 4 presents the torque, humidity, and temperature characterization. The results of the sensor with variations in both temperature and humidity are also presented in Section 4. Final remarks and future works are discussed in Section 5.

## 2. POF Sensors’ Operation Principle

**Figure 1:**
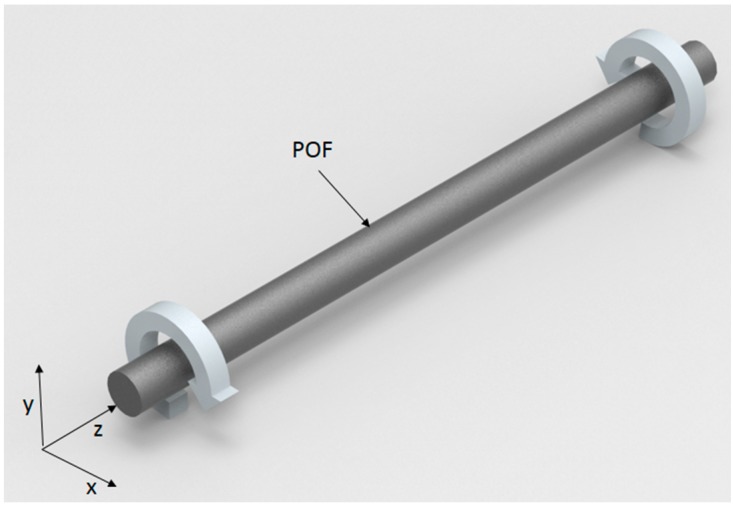
Polymer optical fiber (POF) under torsion stress.

When a fiber is under stress, there is a variation in its refractive index due to the stress-optic effect [27]. This effect is described by a second-rank tensor that represents the changes on the optical indicatrix under predefined stress, as presented in [27]. Such variation in the refractive index leads to variations in the critical angle and the number of modes in the fiber. In the case of fiber torsion presented in Figure 1, the stress tensor of the fiber is presented in Equation (1) [27]:

(1) $$\sigma =\left[\begin{array}{c} 0\\ 0\\ 0\\ \mu \tau x\\ -\mu \tau y\\ 0 \end{array}\right]$$

where μ is the material shear modulus, τ is the torsion angle, and *x* and *y* are the directions of the Cartesian plane defined in Figure 1.

The variation in the refractive index leads to a variation in the POF’s output power. In addition, PMMA POFs show variations in their shear and Young’s moduli when the temperature is changed. Some commercial POFs such as HFBR-EUS100Z (Broadcom Limited, Singapore), which is used in this paper, present a PMMA core with a diameter of 0.98 mm, a cladding of 20 µm thickness made of fluorinated polymer, and a polyethylene coating with a diameter of 2.2 mm that is also characterized by the temperature dependency of its mechanical properties [28]. Besides the variation in the mechanical properties of the fiber, the variation in the *RH* leads to refractive index variation due to water absorption-induced swelling of the PMMA POF [24].

Because the material is affected by both temperature and humidity, a humidity sensor based on this principle will suffer from temperature cross-sensitivity and vice-versa. For this reason, we employed two POFs under torsion stress. Each sensor’s response is a sum of the contribution of the temperature and *RH* variations. Therefore, a system with two variables and two equations was obtained. In order to separate the *RH* and temperature responses, direct differences between the sensors’ equations (considering their sensitivities with respect to temperature and humidity) were applied, as presented in Equation (2). This principle is widely applied to reduce the temperature cross-sensitivity on FBG-based sensors, interferometers, or POF intensity variation-based sensors [5,29,30,31].

(2) $$\left[\begin{array}{l} \Delta RH\\ \Delta T \end{array}\right]={\left[\begin{array}{l} K_{1,RH}\;K_{1,T}\\ K_{2,RH}\;K_{2,T} \end{array}\right]}^{-1}\left[\begin{array}{l} P_{1}\\ P_{2} \end{array}\right]-\left[\begin{array}{l} P_{1,0}\\ P_{2,0} \end{array}\right]$$

where Δ*RH* and Δ*T* are the *RH* and temperature variations, respectively. *K*_1,*RH*_ is the sensitivity of Sensor 1 to the *RH* variation, whereas *K*_1,*T*_ is the sensitivity of Sensor 1 to the temperature variation. The parameters *K*_2,*RH*_ and *K*_2,*T*_ are the sensitivities of Sensor 2 to the *RH* and temperature variations, respectively. *P*_1_ and *P*_2_ are the measured powers of Sensor 1 and 2, respectively. *P*_1,0_ is the initial power of Sensor 1, and *P*_2,0_ is the analogous parameter for Sensor 2. Although generally there may be variations in POF sensors’ sensitivity to temperature or *RH*, it has been demonstrated in [24] that these effects are reduced if the fibers are subjected to strain. Here, because both POFs are submitted to torsional strain, the sensitivity parameters of Equation (2) can be considered as constants. In addition, if Sensors 1 and 2 are connected to the same light source, the effects of power fluctuation from the light source will be compensated for by subtraction from both sensors’ responses through the application of Equation (2), as discussed in [31].

## 3. Experimental Setup

**Figure 2:**
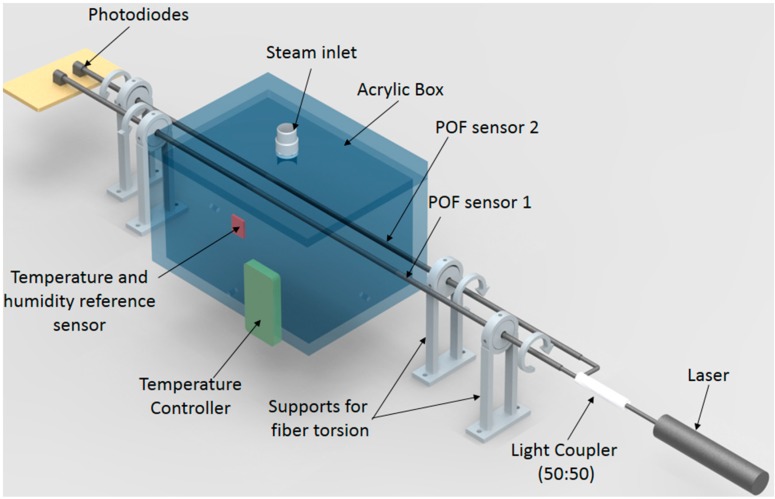
Experimental setup for POF humidity and temperature sensor tests.

Our tests were made with the experimental setup presented in Figure 2. The setup consisted of an acrylic box with an inlet on its top for the injection of steam through an air humidifier. The box also had two holes on its left and right sides for the POF sensors, which were subjected to a constant torque through the four supports presented in Figure 2. Each support had a degree of freedom for rotation around the z-plane (as presented in Figure 1) and a lock mechanism to keep each fiber on the torsion angle applied. The light source was a low-cost laser 3 mW@650 nm, which had its signal divided between both sensors with a 50:50 coupling ratio using a light coupler 1 × 2 IF 562 (Industrial Fiber Optics, Tempe, AZ, USA). Two photodiodes IF-D91 (Industrial Fiber Optics, Tempe, AZ, USA) made the acquisition of the sensors’ power variations in volts (V). Furthermore, the data acquisition was made with the FRDM-KL25Z board (Freescale, Austin, TX, USA) at 200 Hz.

For the tests with temperature variations, the experimental setup presented in Figure 2 was positioned inside a climatic chamber 400/1ND (Ethik Technology, Vargem Grande Paulista, Brazil) with a closed-loop temperature controller (see Figure 2). The reference measurement of temperature and humidity was made with a HTU21D (Measurement Specialties, Hampton, VA, USA) temperature and humidity sensor.

## 4. Results and Discussion

### 4.1. Torque Characterization

A sensor needs a fiber subjected to a constant torque to enhance its sensitivity to temperature and *RH* variations. For this reason, different torques were applied on the fiber to characterize their influence on the POF’s output power. The torque was applied by rotating the fiber in a predefined torsion angle in the directions shown in Figure 2. Because the refractive index variation is directly proportional to the torsion angle [27], it is possible that a higher angle leads to a sensor with higher sensitivity. Furthermore, if the torque is applied in a different direction, it is expected that the power variation will also occur in a different direction, demonstrating sensor polarity; in other words, the sensor can distinguish the increase or decrease of the temperature and humidity. In order to enhance sensor sensitivity, a lateral section was made on the fiber, which consisted of polishing the side of the fiber to remove its cladding and part of the core. The lateral section dimensions are related to the power variation of the sensor and can increase the stress to which the fiber is subjected [32].

**Figure 3:**
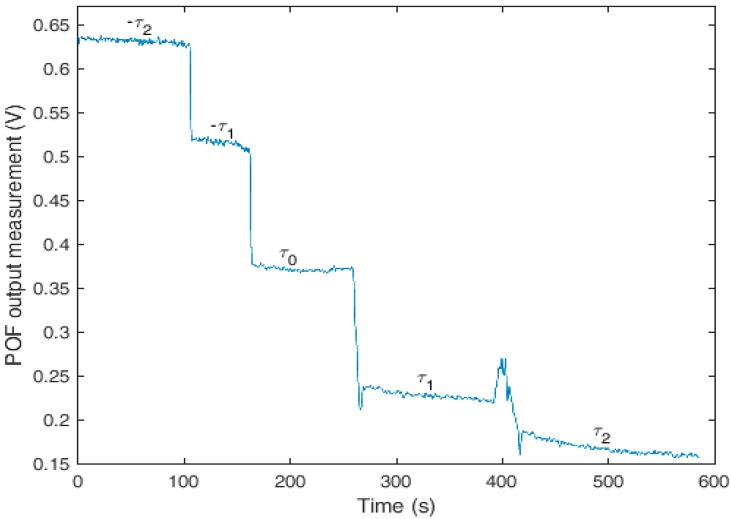
POF response under different torques.

Four different torques were tested: two in the clockwise direction and two in the counterclockwise direction. Figure 3 shows the results of the torque characterization. The torsion angles indicated as *τ*_1_ and −*τ*_1_ were related to approximately the same torsion angle with *τ*_1_ in the clockwise direction and −*τ*_1_ in the counterclockwise direction. The same approach was used for *τ*_2_ and −*τ*_2_, in which the torsion angles *τ*_0_, *τ*_1_, and *τ*_2_ were 0°, 15°, and 45°, respectively. However, the torque applied on *τ*_2_ was lower than that of −*τ*_2_, because the POF’s response would be too attenuated if a torque of the same magnitude as −*τ*_2_ were applied. The region of Figure 3 related to *τ*_0_ depicts the POF’s response without the application of torsion stress. In addition, the peak between *τ*_1_ and *τ*_2_ is related to a torque relaxation in the process of applying the higher torque *τ*_2_.

Referring to Figure 3, the response’s transitions around 280 s and 400 s were related to the process of applying the torque on the fiber and were due to its viscoelastic behavior, which led to a non-constant response of the polymer with stress or strain [33]. The polymer viscoelasticity caused the transient behavior of the response around 280 s and 420 s, at which times there were sudden increases followed by polymer relaxation when the torque was applied, as characterized in [34,35]. It is worth mentioning that polymer relaxation is an intrinsic behavior of the POF used, which occurred for all of the torques tested. Because this effect becomes more evident with strain increases, there was a larger variation in the POF’s response to higher torsion angles. As long as the sensor was isolated from external mechanical disturbances, when the torsion angle *τ*_1_ was applied, there was a stabilization of the voltage after about 150 s. Because such polymer relaxation can be affected by temperature, the temperature was kept at around 20 °C, which led to a lower variation in the polymer’s viscoelastic behavior as demonstrated in [34].

### 4.2. Relative Humidity Characterization

After the torque characterization, two POFs with lateral sections were subjected to humidity characterization. In order to show the influence of the torque’s direction on the response of the sensor, the torques were applied in different directions. On Sensor 1, the torque was applied in the counterclockwise direction, whereas on Sensor 2, the torque was applied in the clockwise direction.

A higher torsion angle was applied on Sensor 2, which subjected it to a higher torque. However, Sensor 2 had a lower lateral section depth than that of Sensor 1. For this reason, the POF’s initial output power for Sensor 2 was lower than that for Sensor 1. The power variation after the torque’s application was higher on Sensor 2 than on Sensor 1.

**Figure 4:**
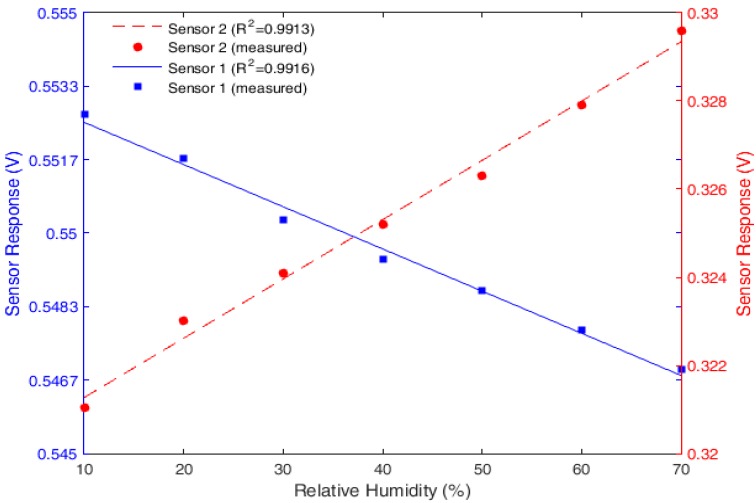
Relative humidity characterization of POF sensors.

Before the *RH* characterization tests, silica gel was inserted in the acrylic box through the steam inlet to reduce the *RH* inside the box (see Figure 2). After the reduction of the *RH*, the output of an air humidifier was positioned on the steam inlet until the *RH* inside the box reached values of about 98%. The same process was repeated three times, and the results obtained for both sensors are presented in Figure 4, in which the blue line and the red dashed line represent the linear fit of Sensor 1’s and Sensor 2’s responses, respectively. Although the test was performed until higher *RH* values were reached, the characterization was limited to the interval of 10–70%. The reason for the analysis on this interval was the lower variations in temperature in that range, which provides the characterization the least influenced by temperature. The temperature in this characterization was 23.64 ± 0.01 °C.

As presented in Figure 4, the torque’s direction led to a variation in sensor polarity. For the counterclockwise torque, the power attenuated when the *RH* increased. As for the torque in the clockwise direction, the opposite effect occurred. The reason for this behavior is related to the change of the direction of each tensor’s component presented in Equation (1). 

### 4.3. Temperature Characterization

The temperature characterization was made by means of positioning the setup presented in Figure 2 in a climatic chamber with closed loop control. The test consisted of increasing the temperature from 24 °C to 44 °C. The reason for this temperature interval is the possibility of a thermal expansion on the POF when the temperature is higher. If this effect occurs, it changes the fiber torsion angle, which leads to a variation in the sensor’s behavior. Furthermore, if the fiber is subjected to higher thermal expansion, it is possible that it will not return to its original shape. Nevertheless, the temperature range applied was within the limits of the comfort regions for microclimate sensing applications, in which such higher thermal expansion may not occur.

**Figure 5:**
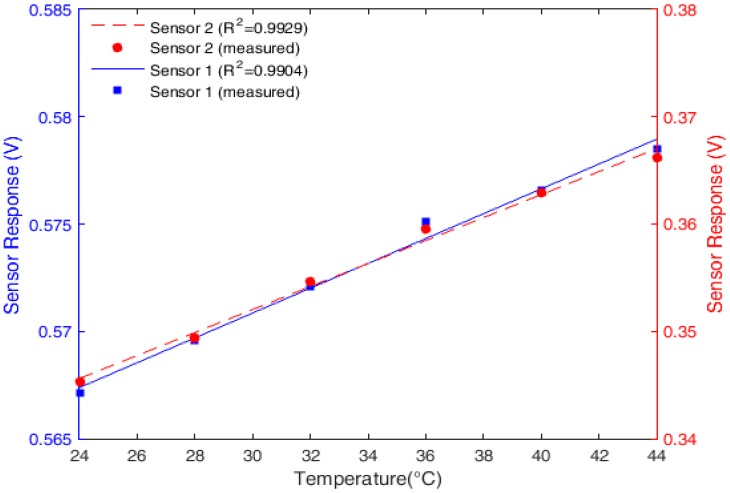
Temperature characterization of POF sensors.

The results of the characterization for both POF sensors and the linear regression of their responses for the temperature test in the range of 24–44 °C are presented in Figure 5. Because the heater used did not have *RH* control, the humidity changed throughout the test. However, the sensor response with respect to the *RH* was already characterized. Therefore, for the temperature characterization without the *RH* cross-sensitivity, the *RH* measured by the reference sensor was applied to the characterization equation obtained in the humidity tests and was subtracted from the response of each sensor in the temperature characterization tests.

In the case of temperature variation, both sensors presented the same behavior: when the temperature increased, the power also increased. This difference in the behavior with temperature and *RH* may have been due to the differences of these parameters on the POF materials, which was related to minor differences between Sensor 1 and 2’s torsion angles. In addition, the POFs’ anisotropy and deviations in the manufacturing parameters, such as pulling force and temperature of the fibers, could have led to differences in the sensors’ behavior. The variation in the humidity may have had different effects on the materials’ mechanical properties, which could also explain the higher sensitivity of Sensor 2 compared with Sensor 1 (see Figure 5). The sensitivity of Sensor 2 to the humidity variations was about 70% higher than that of Sensor 1, whereas the sensitivity to temperature variations was 54% higher than that of Sensor 1.

The higher sensitivity of Sensor 2 to the temperature variations could be related to its higher lateral section depth, which led to an increase in the stress-optic effect variation due to the temperature effects on the POF’s material properties. Because the difference between the sensitivities of Sensors 1 and 2 was higher in the *RH* analysis, the effect of the *RH* on both polyethylene and PMMA could lead to an increase of the modulus of one material and a decrease of the modulus of the other. This could explain the 16 % difference in the ratio between the sensitivities of Sensors 1 and 2 with respect to humidity when compared with the same ratio with respect to temperature.

### 4.4. Simultaneous Measurement of Temperature and Relative Humidity

The test was performed with the setup positioned inside the heater and with the air humidifier output positioned on the steam’s inlet. In this test, the humidity was increased from 5% to 97% without changing the temperature due to operational limitations. However, simultaneous variation of humidity and temperature was obtained in the second part of the test, in which the temperature increased from 24 °C to 38 °C and the humidity dropped to almost 25%. Then, the temperature was increased to about 46 °C and reduced again to about 41 °C, while the humidity had a variation of less than 5%.

**Figure 6:**
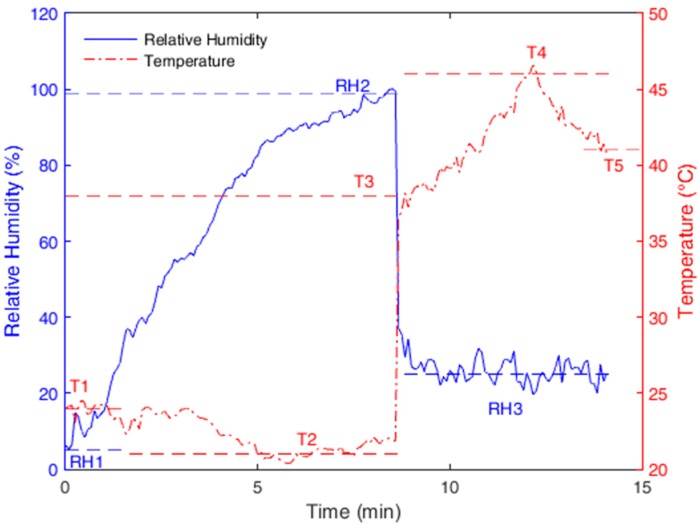
POF sensor’s response to temperature and humidity tests.

Figure 6 shows the results obtained with the POF sensors in the above-described test. The response was obtained by applying Equation (2), in which the coefficients of the equation were obtained from the temperature and *RH* characterizations, which are presented in Table 1.

The blue dashed lines in Figure 6 represent the points or intervals at which the *RH* of the reference sensor was acquired. Point ‘*RH*1’ is the response of the reference sensor at the beginning of the test, and point ‘*RH*2’ is one of the reference sensor’s responses when the humidity reached its maximum value. ‘*RH*3’ relates to the interval of the second part of the test during which the temperature was increased. Because the response time is defined in [23] as the time that the sensor takes to reach the 90% final humidity value (97%), the response time of the proposed sensor is about 5 min (measured between *RH*1 and *RH*2), which is a lower response time than those presented in [23,24] with the additional advantage of a lower cost.

The red dashed lines refer to the intervals of the reference sensor’s response to variations in temperature. ‘*T*1’ is the temperature at the beginning of the test; ‘*T*2’ refers to the decrease in the temperature during the first part of the test; ‘*T*3’ is the temperature measured after the increase of *RH*, rising as long as the humidity decreased; and ‘*T*4’ is the maximum temperature obtained during the test. Finally, ‘*T*5’ is the temperature measured at the end of the test.

The proposed POF sensors are capable of tracking the humidity and temperature variations. Comparing the measured points of the reference sensor with the POF sensors’ responses, the proposed optical fiber sensors showed (with respect to the reference sensor) a root mean squared error (RMSE) of 1.12 °C for temperature and 1.36% for *RH*.

A limitation of these sensors is that greater changes in the sensors’ torques lead to variations in the sensors’ behavior. However, such variations can be reduced if the sensing regions of the fibers are protected with metal coatings or other materials that prevent these changes in the torque from affecting the fiber if it is submitted to an impact or additional strain.

### 4.5. Polymer Optical Fiber Etching to Reduce Humidity Sensor Response Time

Although the proposed sensor is a low-cost solution that is able to measure *RH* with a response time lower than the ones presented in [23,24], it still has a response time higher than that of the etched POFBG presented in [25]. Fiber etching can reduce the polymer Young’s modulus and reduce the sensor’s response time by means of the diameter reduction. In this way, the increase of the POF stress due to fiber diameter reduction improves the humidity sensor’s response time [24].

To reduce further the response time of our sensor, an etching was made on the sensor’s sensitive zone by placing the zone inside a container filled with pure acetone for 4 min. This chemical treatment led to a diameter reduction of the POF sensitive zone of about 20% (0.76 mm). Because it is possible that solvent absorption leads to a molecular chain relaxation that causes the reduction of the Young’s modulus, the effect of fiber etching may result in the reduction of the response time due to two effects. The first is the increase of the stress on the fiber due to the Young’s modulus and diameter reduction, which improves the sensor’s response time, as reported in [24]. The other effect is the increase of the rate of water absorption of the PMMA when its diameter is reduced, as reported in [25].

**Figure 7:**
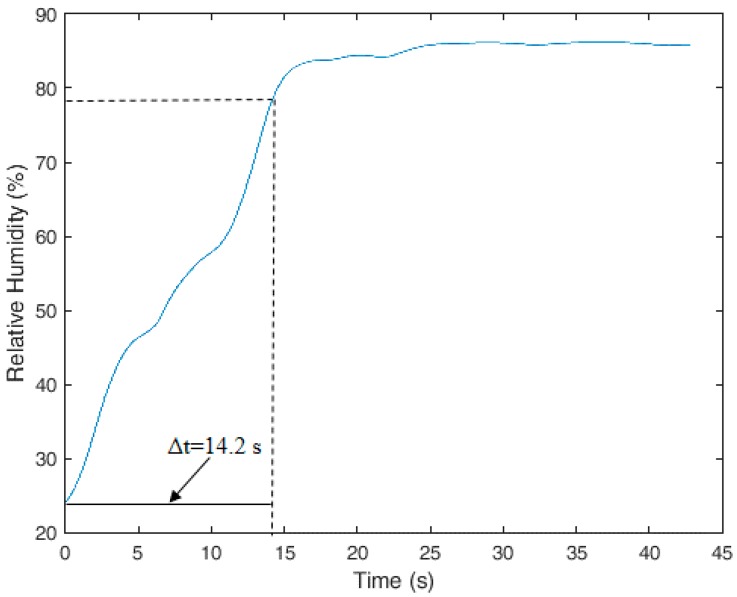
Improved response time with etching on the POF humidity sensor based on the induced stress-optic effect.

The response time improvement of the proposed sensor was demonstrated in the tests with the experimental setup presented in Figure 2, in which the *RH* was increased from 25% to about 85%. The obtained results are presented in Figure 7, where the sensor presented a response time of 14.2 s on the entire range of the test, meaning that for 10% *RH* variation, there was a response time lower than 2.0 s. Such a response time is close to that presented in [25], where a 4.5 s response time for 30% *RH* variation was reported—meaning a response time of about 1.5 s for 10% variation. In addition, the proposed sensor has a lower cost and a higher fiber diameter, which makes the POF more robust and easier to handle. However, etching reduces the sensor’s reproducibility and ease of fabrication, creating a tradeoff between the sensor’s ease of fabrication and its response time. For applications in which response times higher than 2 s are not an issue, it may be preferable to use a humidity sensor without etching treatment, such as the one presented in Section 4.4.

## 5. Conclusions

This paper presented a POF-based sensor system for the measurement of temperature and relative humidity. The sensor is based on the variations in the POF material’s properties when subjected to different temperatures and humidity. In order to measure these variations with a low-cost interrogation system, a predefined torsion angle was applied to both fibers. This stress on the fibers led to output power variations due to the stress-optic effect. Because the stress depended on the fiber material’s properties, the variations in these properties with the temperature and *RH* led to variations in the stress on the fiber, causing variations in the POF’s output power. The POF sensors were characterized relative to the humidity and temperature to obtain an equation that accounted for the contribution of each environmental parameter in the sensors’ responses. After the characterization, the POF sensors were able to measure the temperature and *RH* with an RMSE of 1.12 °C for the temperature and 1.36% for the humidity, with respect to the reference temperature and humidity sensor in the intervals analyzed.

The humidity sensor showed a response time of about 5 min, which is lower than the ones presented in [23,24]. Nevertheless, this response time was further reduced with fiber etching, which led to a response time of 2 s for 10% humidity variation. In addition, it may be possible to reduce the response time with an analysis of the combined effects of the applied torque and etching parameters on the fiber, where an optimization of these parameters can be obtained—a subject that will be investigated in the future. Future works will also include the use of this sensor system to measure microclimate parameters in wearable robotics.

## Figures and Tables

**Table 1:** Parameters applied on the simultaneous measurement of temperature and relative humidity.

Symbol	Parameter Description	Value
*K*_1,*RH*_	Relative humidity sensitivity of Sensor 1	−9.57 × 10^−5^
*K*_1,*T*_	Temperature sensitivity of Sensor 1	5.78 × 10^−4^
*K*_2,*RH*_	Relative humidity sensitivity of Sensor 2	1.34 × 10^−4^
*K*_2,*T*_	Temperature sensitivity of Sensor 2	1.07 × 10^−3^
*P*_1,0_	Initial power of Sensor 1	0.5535
*P*_2,0_	Initial power of Sensor 2	0.3199
